# Supplementary material for: Evaluation of Plasma Lipocalin-2 as a Predictor of Etiology and Severity in Adult Patients with Community-Acquired Pneumonia
Source: Microorganisms. 2023 Apr 28;11(5):1160. doi: 10.3390/microorganisms11051160 (PMC10220602; doi:10.3390/microorganisms11051160)
Supplement: Supplementary file 1 [file microorganisms-11-01160-s001.zip › microorganisms-2321196-supplementary.pdf]

Supplementary material S1

Definitions

Community-acquired pneumonia (CAP) was defined as an acute illness (symptoms lasting for ≤7 days) with the presence of a new infiltrate on a chest radiography associated with two or more of the following signs and symptoms: fever or hypothermia, dyspnoea, cough, sputum production, pleuritic chest pain, altered breath sounds on auscultation. The exclusion criteria were: age <18 years, nosocomial or bronchoaspiratory pneumonia, antibiotic treatment started at the ED >4h before the potential inclusion in the study, hospital admission during the previous 14 days or previous inclusion in the study.

Given the lack of a diagnostic gold standard, pneumococcal pneumonia (P-CAP) was defined by a composite diagnostic variable: detection of *S. pneumoniae* by conventional methods and/or a positive rtPCR-*lytA* in blood, urine or NP swabs (NP cut-off ≥8000 copies/mL) (1). Defined CM were blood culture, good-quality sputum Gram stain, sputum culture or an immunochromatographic test for detection of *S. pneumoniae* antigen in urine.

Invasive pneumococcal disease (IPD) was defined as the presence of *S. pneumoniae* in blood cultures, pleural fluid or cerebrospinal fluid.

Clinical evaluation and follow up

All CAP episodes were evaluated by an infectious diseases' consultant before inclusion. All chest radiographs were reviewed by a radiologist confirming the presence of a new infiltrate.

The following variables were prospectively collected: age, gender, race, vaccination status, immunosuppression (acquired immunodeficiency syndrome, chronic corticosteroid therapy, severe neutropenia, solid or hematopoietic organ transplantation and use of chemotherapy, immunosuppressive agents or biological drugs), clinical, microbiological and laboratory data at admission, evolution, length of stay and outcomes. Variables related to comorbidities (based on Charlson severity index), smoking habit and alcohol abuse were also recorded. Prior antibiotic exposure (in the last 3 months), or acute exposure (last 24 hours before admission) were recorded.

Microbiological tests

Conventional methods included sputum, nasopharyngeal (NP) swab (Deltaswab amies, Deltalab, Rubí, Spain) and blood culture. Sputum samples were processed for Gram stain. Sputum samples were considered of good-quality if they had <10 squamous cells and >25 leukocytes per low-power field. Blood cultures were processed with the BacT-Alert® system (bioMérieux, Marcy-Etoile, France). Identification of isolates were performed by MALDI-TOF (matrix-assisted laser desorption/ionization time-of-flight) mass spectrometry and antimicrobial susceptibility was tested by the microdilution method, following the European Committee on Antimicrobial Susceptibility Testing methods and criteria (EUCAST). Immunocromatopraphic test for *Streptococcus pneumoniae* and *Legionella pneumophila* serogroup I antigen detection in urine was performed (BinaxNOW® assays, Abbott).

Multiplex-PCR for respiratory viruses detention was performed in NP swabs (AllplexTM Respiratory Panel 1, 2 and 3, Seegene®, Seoul, Korean Republic): influenza A

and B virus; respiratory syncytial virus (RSV) A and B; parainfluenza virus (PIV) 1, 2, 3 and 4; coronavirus 229, NL63E and OC43; human rhinovirus (HRV); adenovirus (AdV); metapneumovirus (MPV); bocavirus (HBoV) 1/2/3/4; and enterovirus (HEV).

In patients with no pneumococcal-CAP, serologic methods (on admission and 3–4 weeks thereafter) were used to detect antibodies against *L. pneumophila, Mycoplasma pneumoniae, Chlamydophila pneumoniae* and *Coxiella burnetii*.

References

1. Sidhu M, Wong M, Karstaedt A, Jansen KU, Albrich WC, Mareletsi T, et al. Use of a Rapid Test of Pneumococcal Colonization Density to Diagnose Pneumococcal Pneumonia. Clin Infect Dis. 2011;54(5):601–9.

Supplementary Figure S1. Plasma concentration of LCN2 among patients with bacterial, unknown etiology and viral CAP, excluding patients with bacterial and viral coinfection.

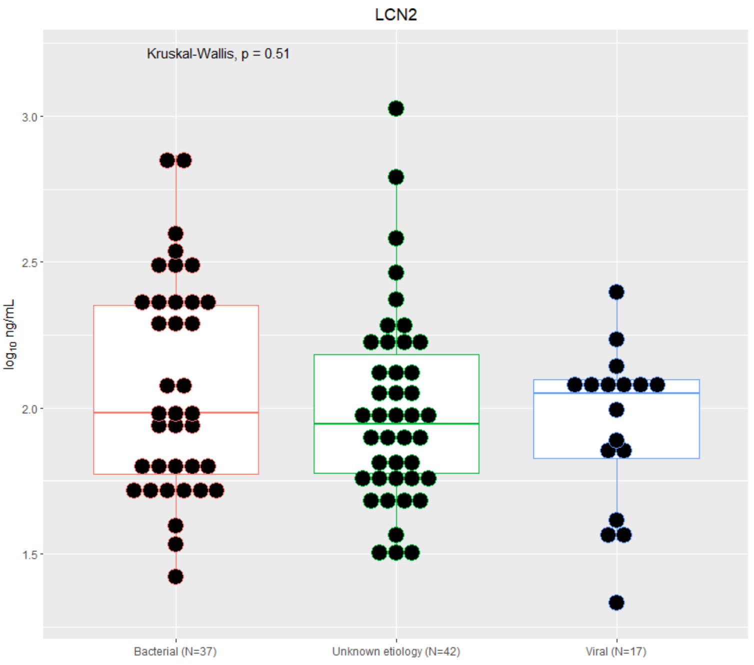

Supplementary Figure S2. Spearman correlation between LCN2 and CRP at admission (A) and between LCN2 and WBC at admission (B).

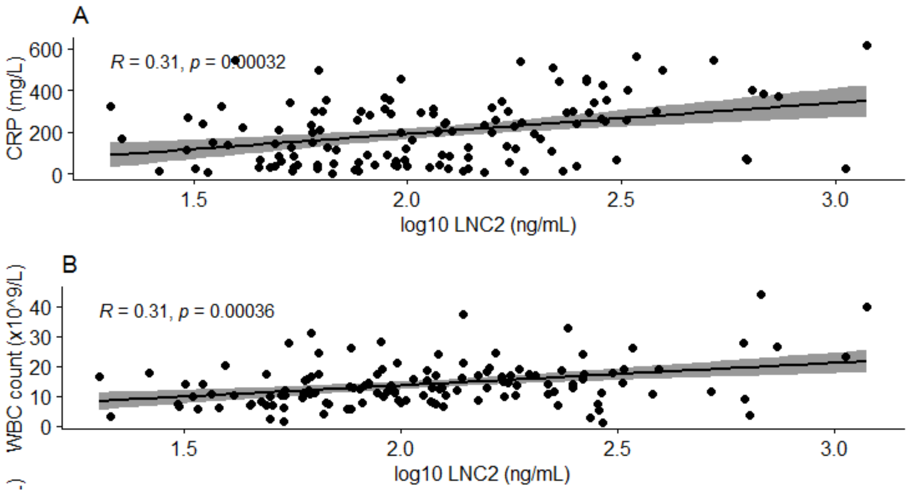

Supplementary Figure S3. Comparison among the paired ROC curves of LCN2, CRP and WBC.

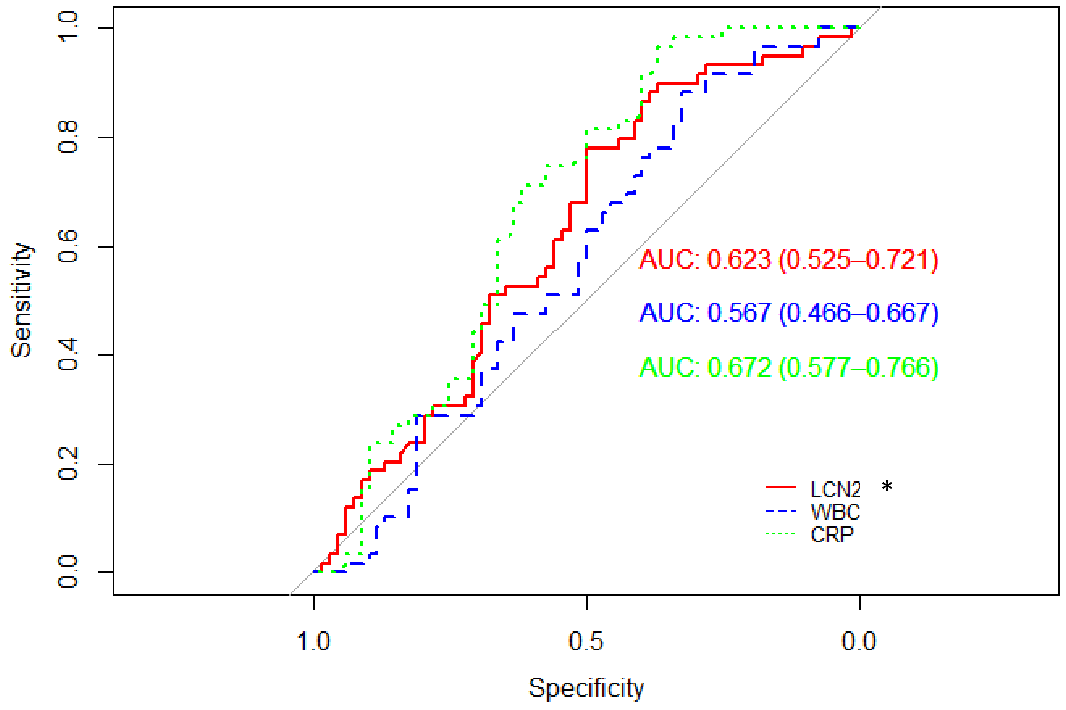

* No statistically significant difference between the AUROC of LCN2, CRP and WBC count to distinguish bacterial and non-bacterial-CAP

Supplementary Figure S4. Comparison among the paired ROC curves of the combination of LCN2 and CRP, LCN2 and WBC or CRP and WBC.

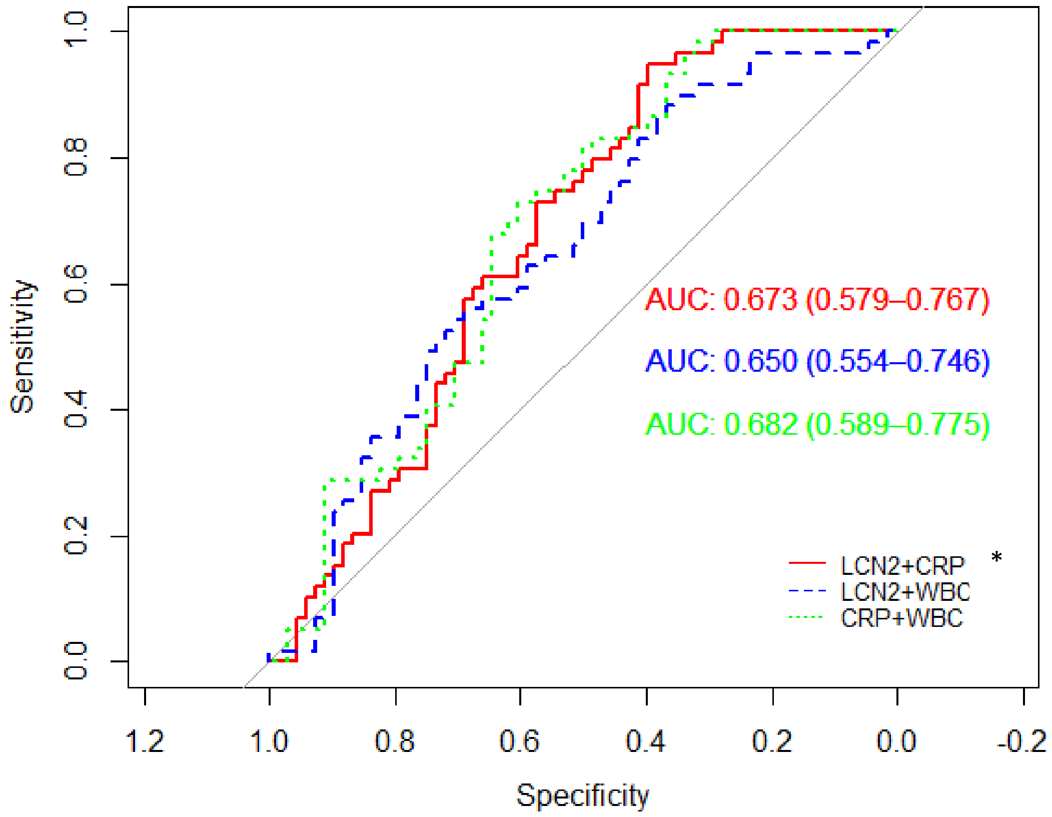

* No statistically significant difference between the AUROC of the different combinations to distinguish bacterial and non-bacterial-CAP
